# A new genomic tool, ultra-frequently cleaving TaqII/sinefungin endonuclease with a combined 2.9-bp recognition site, applied to the construction of horse DNA libraries

**DOI:** 10.1186/1471-2164-14-370

**Published:** 2013-06-01

**Authors:** Agnieszka Zylicz-Stachula, Olga Zolnierkiewicz, Jacek Jasiecki, Piotr M Skowron

**Affiliations:** 1 Department of Chemistry, Division of Theoretical Physical Chemistry, University of Gdansk, Sobieskiego 18, 80-952, Gdansk, Poland; 2 Department of Chemistry, Division of Molecular Biotechnology, University of Gdansk, Sobieskiego 18, 80-952, Gdansk, Poland; 3 Department of Pharmaceutical Microbiology, Medical University of Gdansk, Hallera 107, 80-416, Gdansk, Poland

## Abstract

**Background:**

Genomics and metagenomics are currently leading research areas, with DNA sequences accumulating at an exponential rate. Although enormous advances in DNA sequencing technologies are taking place, progress is frequently limited by factors such as genomic contig assembly and generation of representative libraries. A number of DNA fragmentation methods, such as hydrodynamic sharing, sonication or DNase I fragmentation, have various drawbacks, including DNA damage, poor fragmentation control, irreproducibility and non-overlapping DNA segment representation. Improvements in these limited DNA scission methods are consequently needed. An alternative method for obtaining higher quality DNA fragments involves partial digestion with restriction endonucleases (REases).

We have shown previously that class-IIS/IIC/IIG TspGWI REase, the prototype member of the *Thermus* sp. enzyme family, can be chemically relaxed by a cofactor analogue, allowing it to recognize very short DNA sequences of 3-bp combined frequency. Such frequently cleaving REases are extremely rare, with CviJI/CviJI*, SetI and FaiI the only other ones found in nature. Their unusual features make them very useful molecular tools for the development of representative DNA libraries.

**Results:**

We constructed a horse genomic library and a deletion derivative library of the butyrylcholinesterase cDNA coding region using a novel method, based on TaqII, *Thermus* sp. family bifunctional enzyme exhibiting cofactor analogue specificity relaxation. We used sinefungin (SIN) – an S-adenosylmethionine (SAM) analogue with reversed charge pattern, and dimethylsulfoxide (DMSO), to convert the 6-bp recognition site TaqII (5′-GACCGA-3′ [11/9]) into a theoretical 2.9-bp REase, with 70 shortened variants of the canonical recognition sequence detected. Because partial DNA cleavage is an inherent feature of the *Thermus* sp. enzyme family, this modified TaqII is uniquely suited to quasi-random library generation.

**Conclusions:**

In the presence of SIN/DMSO, TaqII REase is transformed from cleaving every 4096 bp on average to cleaving every 58 bp. TaqII SIN/DMSO thus extends the palette of available REase prototype specificities. This phenomenon, employed under partial digestion conditions, was applied to quasi-random DNA fragmentation. Further applications include high sensitivity probe generation and metagenomic DNA amplification.

## Background

Current rapid technological advances in whole genome DNA sequencing, based on novel or previously existing principles, are gradually replacing established Sanger method variants. Many of these advanced Next Generation Sequencing (NGS) technologies are in widespread use. Examples include 454 pyrosequencing, based on the use of single primer-coated beads, combined with DNA amplification in which luciferase-generated light is emitted upon addition of individual nucleotides to the nascent DNA [1], and rolling circle replication, in which genomic DNA sections are formed into DNA nanoparticles (*Complete Genomics* / *BGI-Shenzhen*) [2]. Another NGS technology, sequencing-by-synthesis (*Illumina* / *Life Sciences*), involves multiple rounds of reversible fluorescent dye-terminator addition to immobilized template by engineered polymerase, imaging, and dye and 3′ blocker removal [3]. Sequencing by ligation / SOLiD technology (*Life Technologies*) uses mismatch-sensitive DNA ligase to join oligonucleotides on complementary template section [4], while ion semiconductor sequencing (*Life Technologies*) employs detection of hydrogen ions produced by DNA polymerization [5]. Single molecule real-time sequencing (*Pacific Biosciences*) is based on fluorescent dye removal upon nucleotide addition [6]. A final example, polony sequencing combines *in vitro* paired-tag library amplification with emulsion PCR, ligation chemistry and automated microscopy [7].

An initial step common to all of these techniques is fragmentation of high molecular weight (HMW) DNA starting material [8]. NGS methods use various HMW materials, including genomic libraries, long-range PCR products, cDNA, and genomic and metagenomic DNA [9]. From this starting material, sequencing libraries and/or PCR matrices are prepared, for use in either NGS or conventional Sanger sequencing. One challenging aspect of high-throughput NGSs is associated with computerized assembly of sequence data when the “bottom-up”, shotgun approach is used for more complex genomes; with sequence repeats that frequently cause gaps in contig assembly are a particular problem. Sequence data fill-in methods, such as long-range PCR and genomic libraries, are thus very useful at this final stage [8]. Libraries are used for physical genome map construction, gene cloning and as a source of direct sequencing templates, which include short genomic fragments up to several thousand bp and P1 phage artificial chromosomes (PACs), bacterial artificial chromosomes (BACs) and yeast artificial chromosomes (YACs) containing large inserts (10–300 kb). Such BAC and YAC clones were recently used to assemble *de novo* an entire synthetic prokaryotic genome and to convert one bacteria species into another [10]. Physical and enzymatic methods, such as low-pressure hydrodynamic shearing [11], sonication [12], atomization [13], nebulization [14], point-sink shearing [15], limited DNAse I digestion [16] and limited restriction endonuclease (REase) cleavage [17], are required to ensure the most unbiased and random possible DNA fragmentation. The first five of these methods are prone to DNA damage, are irreproducible, need frequent calibration and specialized equipment, and are often difficult to automate. Enzymatic methods, including REase digestion, would thus seem to be the methods of choice [8]; however, of more than 300 known naturally-occurring REases that cleave 4-8-bp sequences, all except three CviJI/CviJI* [18-22], SetI [23] and FaiI [23] do so too infrequently (every 256 to 65536 bp) to easily generate complete coverage with randomly overlapping fragments, even under partial digestion conditions. An alternative approach, quasi-random fragmentation, involves the application of the enzyme mixture NEBNext dsDNA Fragmentase. In this method dsDNA breaks are produced by the concerted action of two enzymes, with one enzyme randomly nicking dsDNA, and the other recognizing the nicked site and cutting the DNA strand opposite the nick [23]. Another drawback of REase-based approaches is that the distribution of REase recognition sites is variable within different genes, DNAs of different GC content, DNA regions and genomes [24], requiring the construction of multiple libraries with different enzymes. Creation of a set of effective enzymatic molecular tools would consequently help overcome these problems, thereby speeding up the implementation of genomic research projects.

We have previously reported that TspGWI, a member of our newly-designated *Thermus* sp. family of bifunctional REases-MTases [25-31], exhibits a novel type of substrate specificity change causing much more frequent cleavage. This feature could be useful for improving genomic technologies [32]. The observed specificity change [32] is induced by the replacement of the enzyme cofactor SAM with its analogue, SIN, which causes a change in REase cleavage frequency that is statistically equivalent to a 5-bp to 3-bp recognition site shift. The new TaqII/SIN/DMSO “molecular scissors” presented in this paper are potentially very useful for generating quasi-random genomic libraries, as there are only five other high-specificity enzyme that possess similarly frequent DNA cleavage properties: CviJI/CviJI*, FaiI, SetI, TspGWI/SIN and NEBNext dsDNA Fragmentase. In addition to its use for library construction and sequencing, new ultra-frequent DNA fragmentation technology based on the unique “affinity star activity” (i.e., relaxed sequence recognition) of some of the *Thermus* sp. family enzymes may be useful for other cloning applications.

In this paper, we describe a second case (after TspGWI/SIN) of this unusual type of REase specificity relaxation, in which a 6-bp recognition site was replaced by the statistical equivalent of a 2.9-bp recognition site. In addition to its basic research aspect, this discovery has important practical applications for the fields of genomics, metagenomics and biotechnology. To demonstrate its usefulness, we applied this technology to the construction of *Equus caballus* (horse) genomic and cDNA libraries. We also used this tool to generate butyrylcholinesterase coding segment deletion derivatives, which in a subsequent study (manuscript in preparation) was used to aid cloning and expression of a biologically active enzyme.

## Results and discussion

### Optimization of the synergistic effect of SIN, reaction pH, salt and DMSO concentrations on the maximum “affinity star” (affinity star) of TaqII specificity

Our previously published preliminary results suggested that TaqII REase exhibits pronounced star activity, which can be further stimulated by SIN. In those earlier studies, however, TaqII affinity star specificity was not determined, nor were reaction conditions of this phenomenon evaluated in great detail [29,30,32]. To investigate basic research aspects of this specificity and to adapt TaqII REase and its affinity star variant for practical use in recombinant DNA technology, we studied reaction parameters to determine those required to achieve: (*i*) the lowest minimum affinity star activity maintaining reasonable cleavage activity with 5′-GACCGA-3′ cognate specificity [29] and (*ii*) maximum stimulation of TaqII specificity transition towards ultra-frequent cleavage.

In previous study [32], we had observed that both TspGWI and TaqII were affected by SAM and SIN; however, the SIN stimulatory/relaxation effect of TaqII, although evident, manifested itself much more slowly and to a lesser extent. To enhance the rate of the specific SIN effect on TaqII, in this study we therefore explored other reaction conditions, such as pH, salt concentration and the presence of DMSO. Because preliminary experiments showed that DMSO was highly stimulatory compared with other organic solvents (not shown), we investigated it further. In addition, our experiments revealed a somewhat unexpected phenomenon: pH, salt and DMSO concentrations effects were not simply additive with respect to the SIN stimulatory/relaxation effect, but were instead more intricately intertwined. More elaborate experiments were therefore needed to pinpoint minimum and maximum affinity star digestion conditions.

**Figure 1:**
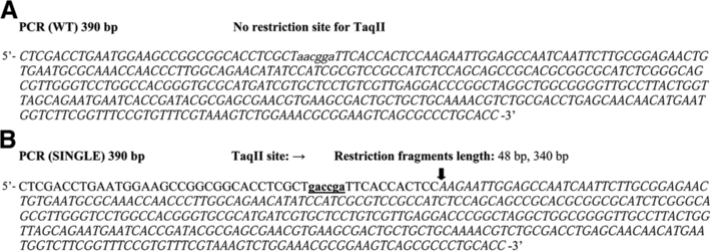
**PCR fragment DNA substrates.** Putative recognition sequence of TaqII is in bold and underlined. Arrows mark the cleavage. The restriction fragments lacking TaqII recognition sequence are in italics. (**A**) PCR fragment without a TaqII site. (**B**) PCR DNA fragment with a single 5′-GACCGA-3′ site.

**Figure 2:**
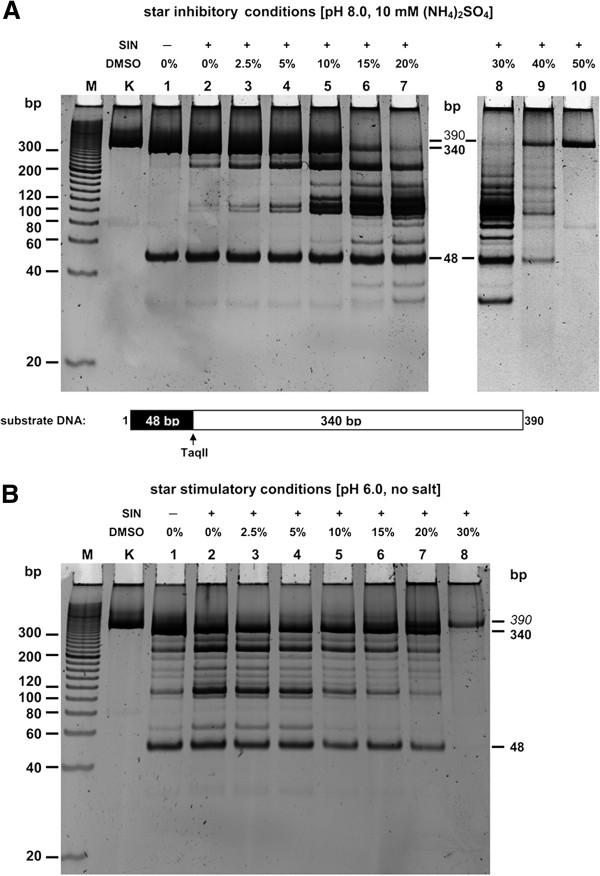
**Comparison of affinity star minimizing and maximizing reaction conditions on SIN/DMSO-induced TaqII REase specificity change.** (**A**) Effect of SIN and DMSO under star minimizing conditions. The influence of SIN and DMSO on TaqII activity was evaluated in reaction conditions minimizing star activity, as we reported previously [29]. 0.3 μg (1.2-pmol GACCGA recognition sites) PCR(SINGLE) substrate was digested with 6 pmol TaqII in the reaction buffer: 40 mM Tris–HCl, pH 8.0, at 65°C, 10 mM (NH_4_)_2_SO_4_, 10 mM MgCl_2_, 1 mM DTT, BSA 100 μg/ml, 100 μM SIN in the DMSO concentration range from 0 to 50% for 16 h at 65°C. Lane M, Sigma PCR 20-bp Low Ladder (selected bands marked); lane K, undigested PCR fragment; lanes 1–9, digested PCR fragment in the presence of increasing DMSO concentrations: lane 1, 0% DMSO; lane 2, 2.5%; lane 3, 5%; lane 4, 10%; lane 5, 15%; lane 6, 20%; lane 7, 30%; lane 8, 40%; lane 9, 50%. (**B**) Effect of SIN and DMSO under star stimulating conditions. The influence of SIN and DMSO on TaqII activity was evaluated in reaction conditions stimulating star activity, as we reported previously [29]. The reaction was conducted as described in **A** in the reaction buffer: 40 mM Tris–HCl, pH 6.0, at 65°C, 10 mM MgCl_2_, 1 mM DTT, BSA 100 μg/ml, 100 μM SIN. Lanes M, K and 1–7 are as described above in **A**.

As a starting point, we used our previously published TaqII star inhibitory/ stimulatory buffer compositions [29]: (1) 40 mM Tris–HCl (pH 8.0 at 65°C), 10 mM (NH_4_)_2_SO_4_, 10 mM MgCl_2_, 1 mM DTT and BSA (100 μg/ml) and (2) 40 mM Tris–HCl (pH 6.0 at 65°C), 10 mM MgCl_2_, 1 mM DTTand BSA (100 μg/ml). Both reaction buffer variants were supplemented with a 100 μM saturating concentration of SIN (not shown) and optimized for DMSO. To precisely determine tested reaction factors, confirm they induced the same specificity and simplify interpretation of electrophoresis results, a custom 390-bp PCR(SINGLE) fragment with a single (→) 5′-GACCGA-3′ site was used as a DNA substrate for cleavage reaction analysis. This substrate, with the TaqII recognition sequence, was obtained using PCR with a forward mutagenic primer, (Figure 1). We tested the effect of different DMSO concentrations, ranging from 0 to 50%, under the two radically different reaction buffer conditions described above. Cleavage reactions were performed for 16 h under enzyme saturating conditions (5:1 molar ratio of enzyme to 5′-GACCGA-3′ sites), designed to ensure TaqII enzyme was not a limiting factor in the initial experiment (not shown). Our results were interesting and unexpected: maximum star activity was observed with 20-30% DMSO at pH 8.0, which in the absence of DMSO inhibited TaqII star activity (Figure 2A; lanes 7–8). We previously found that pH 6.0 was highly stimulatory for natural and SIN-induced TaqII specificity (star) changes [29]; however, at pH 8.0, the addition of DMSO radically changed the TaqII response to SIN. Although the banding pattern observed at pH 6.0 and 8.0 in the presence of SIN and DMSO pointed to the same affinity star recognition site specificity, a pH of 8.0 stimulated the enzyme and relaxed its specificity much more so than did pH 6.0 (Figure 2). To determine the set of conditions leading to a fully relaxed TaqII recognition sequence and to practically apply this phenomenon to quasi-random genomic library construction, in addition to other reasons further experiments were performed at pH 8.0. Although we did not investigate the chemical nature of the pH- and DMSO-dependent SIN effect, we suggest that the results may be due to alternations in the protonation state of the pentose-attached SIN, side chain which contains two amino groups and a carboxyl group in close proximity to one another. These charge fluctuations may affect the interaction of SIN bound to the TaqII allosteric protein motif and cause subtle differences in active protein conformation, which are enhanced by the presence of DMSO.

Bearing in mind the significant influence of ionic strength on cognate TaqII and TaqII star activity ( [29]; this work), we performed a series of TaqII cleavage reactions with variable concentrations of ammonium sulfate in the pH-optimized reaction buffer to ascertain maximum affinity star stimulatory conditions. We chose ammonium sulfate as the salt component because preliminary experiments indicated it had a generally slightly higher cognate cleavage stimulatory effect than the commonly used NaCl at equivalent ionic strength. Ammonium sulfate also appears to stabilize TaqII to a greater extent ( [29]; unpublished results).

**Figure 3:**
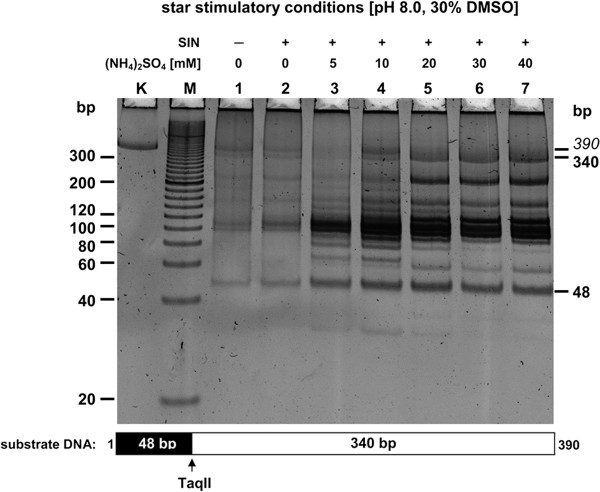
**The effect of ionic strength on TaqII REase at pH 8.0 in the presence of the SIN/DMSO combination range, maximally stimulating the specificity change.** 0.3 μg (1.2-pmol GACCGA recognition sites) PCR(SINGLE) substrate was digested with 6 pmol TaqII in 50 μl of the reaction buffer: 40 mM Tris–HCl, pH 8.0, at 65°C, 10 mM MgCl_2_, 1 mM DTT, BSA 100 μg/ml, 100 μM SIN, 30% DMSO for 16 h at 65°C. Lane K, undigested PCR fragment; lane M, Sigma PCR 20-bp Low Ladder (selected bands marked); lanes 1–7: digested PCR fragment; lane 1, without SIN and (NH_4_)_2_SO_4_; lane 2, with SIN and without (NH_4_)_2_SO_4_; lanes 3–7 contain SIN and increasing concentrations of (NH_4_)_2_SO_4_: xlane 3, 5 mM (NH_4_)_2_SO_4_; lane 4, 10 mM; lane 5, 20mM; lane 6, 30 mM; lane 7, 40mM.

The maximum affinity star activity in the tested 0–40 mM ammonium sulfate concentration range was obtained at 10 mM, which was much higher than that observed between 0 and 5 mM (Figure 3; lanes 3–4). Again, this is atypical, as most REases become more prone to star activity, when the ionic strength is decreased [33]. The presence of small amounts of salts such as ammonium sulfate contributing both ammonium and highly charged sulfate ions, may stabilize interaction between TaqII and SIN; these latter molecules apparently form a complex with different properties than those of the natural TaqII-SAM complex.

In summary, maximum TaqII affinity star activity, as established under carefully controlled substrate and reaction conditions, took place in 40 mM Tris–HCl (pH 8.0 at 65°C), 10 mM (NH_4_)_2_SO_4_, 10 mM MgCl_2_, 1 mM DTT, BSA (100 μg/ml), 100 μM SIN and 30% DMSO. Because 30% DMSO significantly hindered agarose gel electrophoresis and gel isolation of the resulting longer restriction fragments, it was less practically suitable than 20% DMSO. Reaction mixtures obtained using 30% DMSO required proteinase K treatment, phenol extraction and ethanol precipitation prior to electrophoresis. These procedures were necessary to prevent diffuse gel bands and eliminate macromolecular complexes, formed when larger DNAs are digested following cleavage by TaqII/SIN/DMSO, and which barely move on the electrophoretic gel. Because it was more suitable for the cleavage of high molecular mass DNA substrate the 20% DMSO concentration was consequently chosen for further experiments.

### Independence of SIN/DMSO-induced affinity star TaqII DNA cleavage from cognate TaqII recognition sequence presence

**Figure 4:**
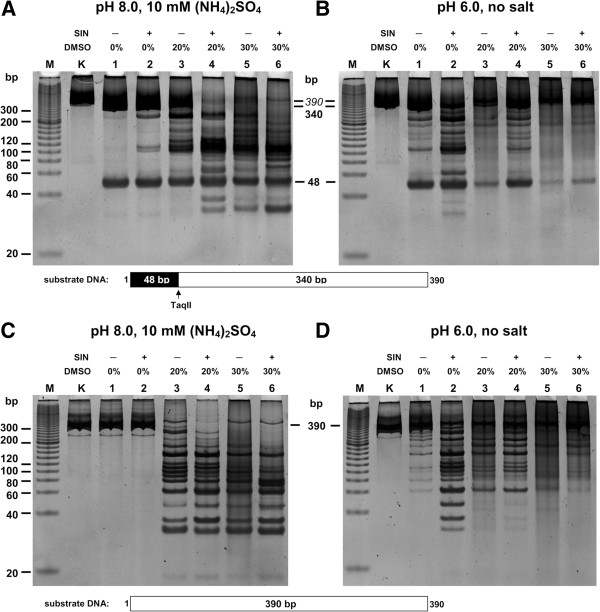
**Comparison of combined SIN/DMSO effect on TaqII REase cleavage patterns of custom PCR substrates with and without a cognate recognition sequence.** (**A**) and (**B**) Digestion of PCR substrate with a single 5′-GACCGA-3′ sequence. 0.3 μg (1.2-pmol GACCGA recognition sites) PCR(SINGLE) substrate was digested with 6 pmol TaqII at pH 8.0 in the presence of 10 mM (NH_4_)_2_SO_4_, (panel **A**) or at pH 6.0, no salt added (panel **B**), for 16 h at 65°C. (**A**) Lane M, Sigma PCR 20-bp Low Ladder (selected bands marked); lane K, undigested PCR fragment; lanes 1–6, PCR fragment digested with TaqII: lane 1, without SIN and DMSO; lane 2, with SIN, no DMSO; lane 3, 20% DMSO only; lane 4, with SIN and 20% DMSO; lane 5, 30% DMSO only; lane 6, with SIN and 30% DMSO. (**C**) and (**D**) Digestion of PCR substrate devoid of the TaqII recognition sequence. All the reactions were performed as in Panels **A** and **B**, except that the PCR(SINGLE) substrate was replaced by PCR(WT) substrate, containing no cognate TaqII recognition site.

Although TspGWI and TaqII are closely related with respect to amino acid sequence properties and belong to the same *Thermus* sp. enzyme subfamily, they exhibit marked differences in cognate site arrangement preferences [27,29,30,32]. In contrast to TspGWI, which prefers the presence of two cognate sites in a DNA substrate [27,32], TaqII REase can cleave a single canonical 5′-GACCGA-3′ site regardless of whether SAM or SIN is present in the reaction buffer (Figure 4A,B; lane 1) [29]. The TaqII cleavage pattern observed is strongly dependent on the reaction buffer used. When we used the TaqII star inhibitory condition (pH 8.0, 10 mM (NH_4_)_2_SO_4_, no SIN) determined from our earlier study [29], TaqII REase cleavage of a single site substrate (→) (Figure 1B) was efficient and yielded the expected 48-bp DNA fragment (Figure 4A; lane 1). The addition of SIN to the reaction buffer only marginally stimulates TaqII star activity at pH 8.0 and then only when the canonical TaqII site was present in the DNA substrate (Figure 4A; lane 2).

The analogous DNA fragment lacking a cognate TaqII recognition sequence (Figure 1A) was not cleaved at pH 8.0, either in the presence or absence of SIN (Figure 4C; lanes 1–2). Under star stimulatory conditions (at pH 6.0), however, TaqII REase relaxation was clearly noticeable in the specificity of DNA recognition, even in the absence of SIN (Figure 4D, lane 1). The addition of the cofactor analogue strongly stimulated TaqII affinity star activity, as demonstrated by the appearance of multiple additional bands (Figure 4D; lane 2).

At pH 8.0 the influence of DMSO on TaqII REase was similar for cognate site (+) and (−) DNA substrates. This organic solvent strongly stimulated TaqII star activity, regardless of whether the TaqII canonical site was present or absent (Figure 4A,C). The strongest effect was observed with 20-30% DMSO in combination with SIN. At pH 6.0, however, the addition of DMSO exerted opposite inhibitory effect, decreasing both cognate TaqII and SIN-induced TaqII affinity star activities (Figure 4B,D). The above results, demonstrated using model DNA fragments, were confirmed through practical biotechnological applications: horse genomic DNA and butyrylcholinesterase cDNA library construction. The PCR product of a 1841 bp long cDNA fragment, corresponding to the butyrylcholinesterase intronless gene and its short (5 and 27 bp) flanking sequences was fragmented in the predicted fashion, analogous to the PCR model described above.

### Determination of TaqII affinity star recognition sequences and cleavage site

**Figure 5:**
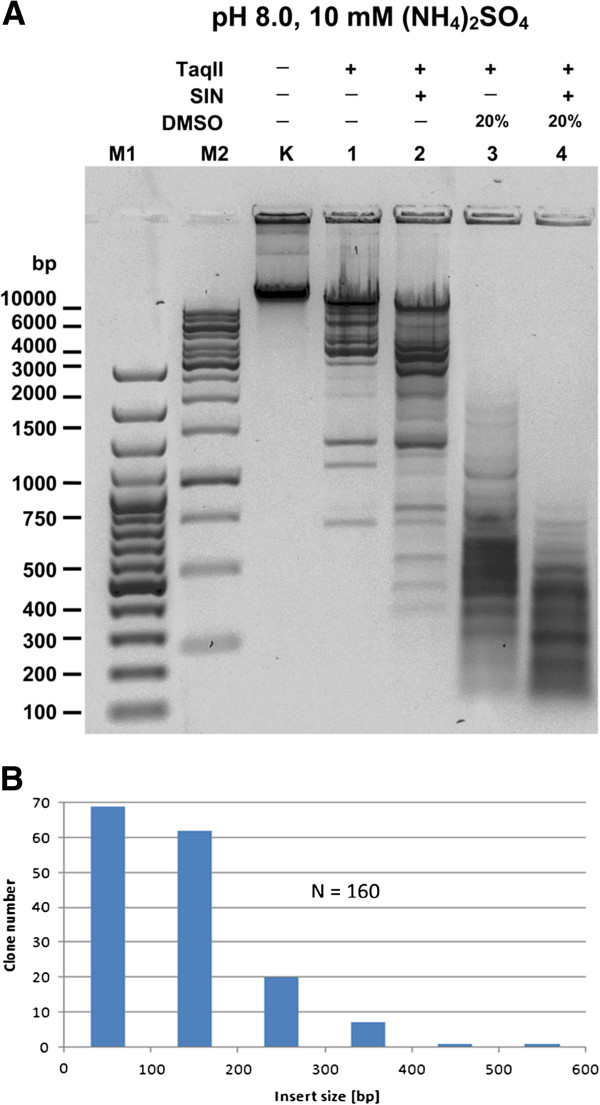
**Cleavage of λ DNA (48,502 bp) under TaqII specificity changing conditions.** (**A**) Complete TaqII cleavage pattern λ DNA under affinity star maximizing conditions (Methods). Samples of 1 μg λ DNA (=0.32 pmol recognition sites), digested under various conditions, were electrophoresed on 1% agarose/TBE gel. The TaqII/SIN/DMSO cleavage products were subsequently shotgun cloned to determine affinity star specificity (Methods). Lane M1, Fermentas 100 bp DNA Ladder (selected bands marked); lane M2, Fermentas 1 kb DNA Ladder (selected bands marked); Lane K, undigested λ DNA; lanes 1–4, λ DNA digested with TaqII: lane 1, without SIN and DMSO; lane 2, with SIN, no DMSO; lane 3, with 20% DMSO, no SIN; lane 4, with SIN and 20% DMSO. (**B**) Insert size distribution of 160 clones randomly selected from TaqII SIN/DMSO-generated λ DNA library in pUC19 vector (see Methods, Figures 2, 3 and 6). The average insert size of the library was estimated to be 160 bp.

To determine the recognition site specificity and cleavage positions of TaqII affinity star activity induced by SIN/DMSO, we performed shotgun cloning of the digestion products of bacteriophage lambda (λ) DNA (Figure 5). TaqII cleavage reactions were carried out using a recombinant enzyme in the presence of affinity star stimulating factors: either a combination of 100 μM SIN/20% DMSO (acting synergistically), or 100 μM SIN alone. The generated restriction fragment ends were repaired with T4 DNA Polymerase/dNTPs, and cloned into the SmaI site of a pUC19 vector [17]. To identify vector-insert junctions, we sequenced 160 randomly chosen clones.

**Figure 6:**
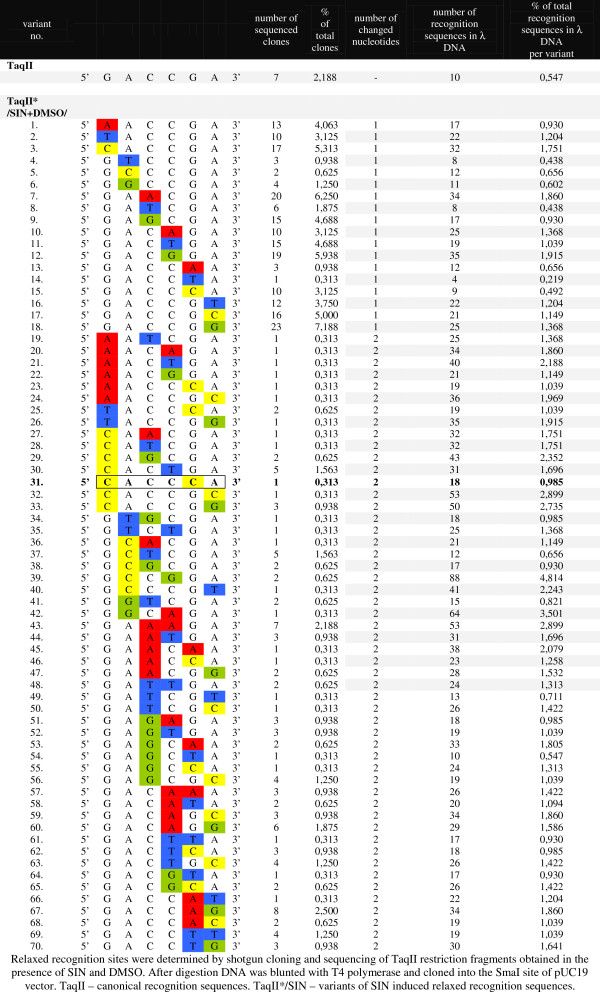
**Specificity change of TaqII REase in the presence of SIN and DMSO.** Relaxed recognition sites were determined by shotgun cloning and sequencing of TaqII restriction fragments obtained in the presence of SIN and DMSO. After digestion DNA was blunted with T4 polymerase and cloned into the SmaI site of pUC19 vector. TaqII – canonical recognition sequences. TaqII*/SIN – variants of SIN induced relaxed recognition sequences.

Analysis of the resulting sequence data revealed that in the presence of SIN/DMSO with TaqII in molar excess over recognition sites (enzyme saturating conditions), the REase recognized and cleaved at least 70 variants containing altered bases of the canonical 5′-GACCGA-3′ sequence (Figure 6). Changes in the restriction site involved one or two bp in the canonical 6-bp DNA sequence (Figure 6). Interestingly, no fixed “core”-invariable recognition sequence was identified, and variants with up to two base departures from the canonical sequence, regardless of the location, were recognized (Figure 6). No variants exhibited changes in both first and second positions, nor in both second and fifth positions. The fact that adenine, which is methylated by TaqII methyltransferase activity could be replaced by any of the other bases is particularly fascinating. This implies that the enzyme was no longer flipping the base into the methyltransferase binding pocket [34], a process that contributes a significant amount of the binding energy for normal Type IIG recognition. In the presence of SIN without DMSO, however, preferred kinetic specificity changes favored single-bp departure canonical site variants, although 2-bp departures from the canonical TaqII site were also present (not shown). It can thus be concluded that DMSO acts as an enhancer of the SIN-specific relaxation effect.

Under standard (star minimum) conditions, TaqII canonical 5′-GACCGA-3′ sequences would be expected to be cleaved on average every 4096 bp [29]. SIN/DMSO-induced TaqII affinity star activity resulted in extremely frequent cleavage approaching that of the most frequent cutters, i.e., CviJI/CviJI* (recognition site: 5′-RGCY-3′/5′-GC-3′), SetI (5′-ASST-3′) and FaiI (5′-YATR-3′) [18-20,23]. Counting, TspGWI/SIN 3-bp specificity, TaqII/SIN/DMSO would thus be only the fifth frequent cutter available out of all known REase prototype specificities. Based on 71 variants of the 6-bp canonical site being detected (including cognate site 5′-GACCGA-3′), complete DNA digestion by the affinity star TaqII should theoretically lead to cleavage approximately every 57.7 bp (4096/71), equivalent to a 2.9 bp long recognition site. As in the case with TspGWI/SIN/DMSO and the 2/3-bp cutter CviJI/CviJI* [18,20,35], however, complete digestion does not take place. One explanation for this behavior, common to all these enzymes, might be steric limitations imposed by cleavage of the very short DNA substrates dominating the reaction during the course of digestion [36]. Even under partial digestion conditions, however, very frequent cutters are still useful for genomic library preparation, yielding a quasi-random accumulation of DNA sequences ( [20,21,37]; this work) they are also of value in other cloning technologies, including ultrasensitive DNA; labeling/amplification [20], highest resolution restriction mapping [19,20], RFLP, single-copy gene amplification, detection/identification of non-cultured pathogenic microorganisms [19,22] and for increasing the limited pool of commercially available Type II REases specificities.

### TaqII/SIN/DMSO cleavage of complex bacterial genomes

**Figure 7:**
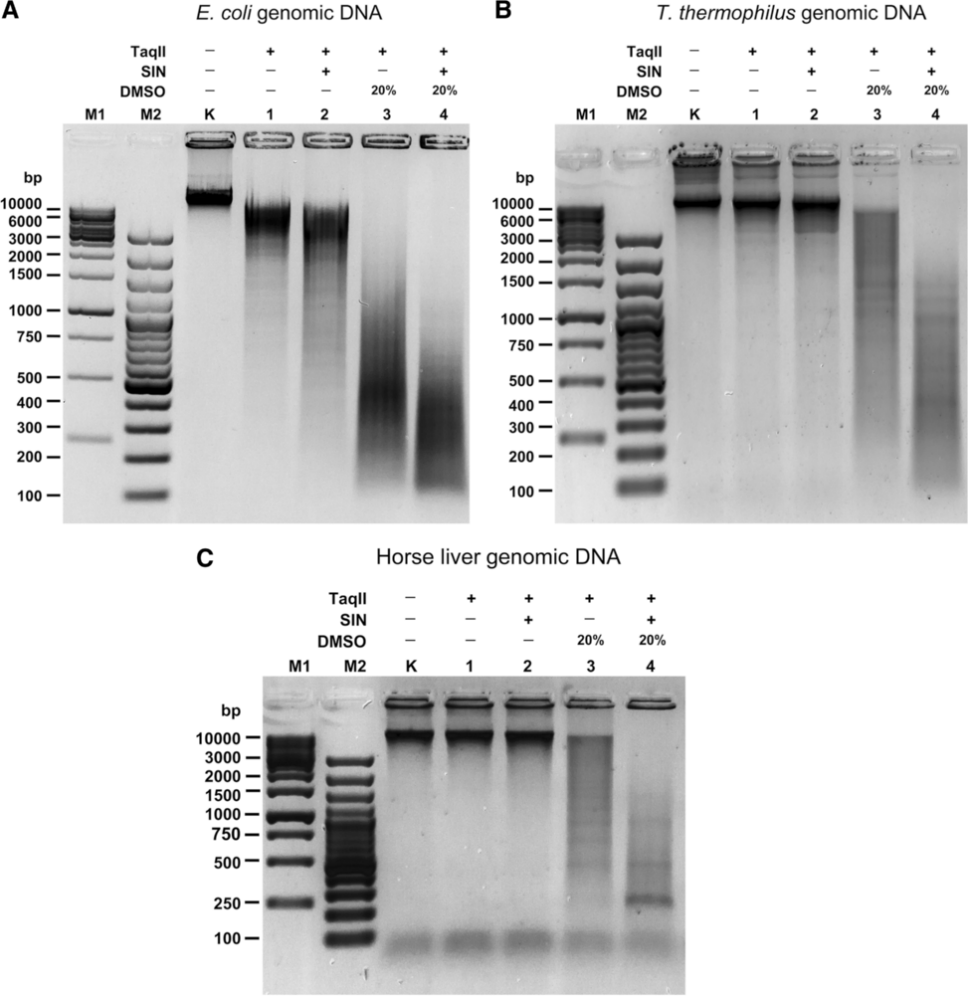
**Digestion of complex bacterial genomes with various sizes and GC contents.** TaqII affinity star cleaved 0.5 μg bacterial (*E. coli* and *T. thermophilus*) genomic DNA samples were electrophoresed on 1.5% agarose/TBE gel. Cleavage (maximum extend) was carried out under specificity change stimulatory conditions (see Methods Figures 2, 3 and 6). (**A**) TaqII affinity star cleavage of *E. coli* genomic DNA (51% GC, 4.6 Mb [GeneBank CP000948]). Lane M1, Fermentas 1 kb DNA Ladder (selected bands marked); lane M2, Fermentas 100 bp DNA Ladder (selected bands marked); Lane K, undigested genomic DNA; lanes 1–4, DNA digested with TaqII: lane 1, without SIN and DMSO; lane 2, with SIN added, no DMSO; lane 3, no SIN, 20% DMSO; lane 4, with SIN and 20% DMSO. (**B**) TaqII affinity star cleavage of *T. thermophilus* genomic DNA (69.4% GC, 1.89 Mb [GenBank AE017221]). Reactions were conducted as in Panel **A**, except that *T. thermophilus* genomic DNA was used as substrate. (**C**) TaqII affinity star cleavage of horse genomic DNA (51% GC, 2.47 Gb [GenBank AAWR02000000]). Reactions were conducted as in Panel **A**, except that horse genomic DNA was used as substrate.

To determine desirable reaction conditions and test the utility of TaqII/SIN/DMSO for HMW DNA digestion, two bacterial genomes with different GC contents were selected: *Escherichia coli* (51% GC; 4.6 Mb) and *Thermus thermophilus* HB27 (69.4% GC; 2.13 Mb) [38]. Cleavage reactions were performed using the optimized reaction buffer discussed above pH 8.0 with (NH_4_)_2_SO_4_, SIN and 20% DMSO (Figure 7A,B). When digested under enzyme saturating conditions TaqII/SIN/DMSO activity was able to easily fragment HMW DNA into fragments less than approximately 500-bp long, rather than clear superimposed bands due to highly biased site preference, a “smear” was observed on the electrophoretic gel. TaqII/SIN/DMSO may therefore be a useful tool for quasi-random fragmentation of complex genomic DNA for genomic library preparation. The average restriction fragment size obtained in an analogous experiment comparing digestion of *E. coli* and *T. thermophilus* genomes was slightly different, with larger sizes observed in the case of *T. thermophilus* (Figure 7A,B; lanes 4). This may have been due to the less intense distribution of TaqII affinity star sites in the high GC-content DNA or impaired binding of TaqII to such substrate DNA under affinity star conditions.

### TaqII/SIN/DMSO cleavage and library construction of eukaryotic (horse liver) genomic DNA and butyrylcholinesterase cDNA

**Figure 8:**
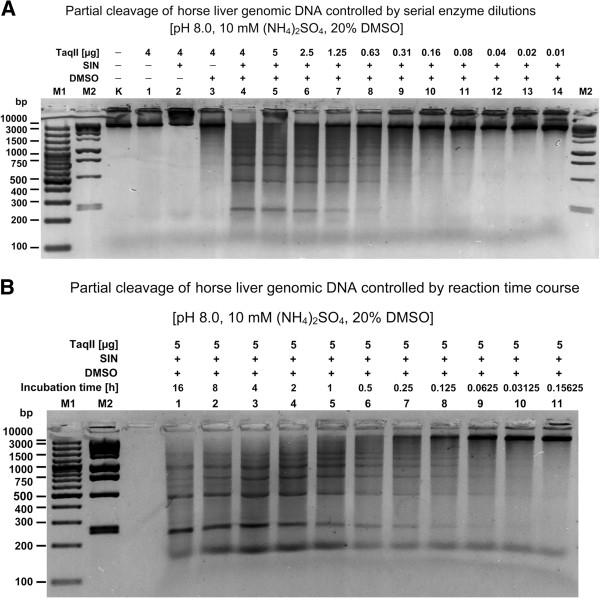
**Digestion of highest complexity genomic DNA (*Equus caballus*) with TaqII/SIN/DMSO for BAC library construction.** TaqII affinity star cleaved 1 μg horse liver DNA was electrophoresed on 1.5% agarose/TBE gel. Cleavage was carried out as described in Methods. (**A**) Partial digestion controlled by serial enzyme dilutions. Lane M1, Fermentas 100 bp DNA Ladder (selected bands marked); lane M2, Fermentas 1 kb DNA Ladder (selected bands marked); Lane K, undigested genomic DNA; lanes 1–4, DNA digested with 4 μg (32 pmols) of TaqII (Methods): lane 1, without SIN and DMSO; lane 2, with SIN, no DMSO; lane 3, no SIN, 20% DMSO; lane 4, with SIN, 20% DMSO; lanes 5–14, DNA digested with twofold dilutions of TaqII for 3 h at 65°C in the presence of SIN/DMSO: lane 5, 5 μg; lane 6, 2.5 μg; lane 7, 1.25 μg; lane 8, 0.63 μg; lane 9, 0.31 μg; lane 10, 0.16 μg; lane 11, 0.08 μg; lane 12, 0.04 μg; lane 13, 0.02 μg; lane 14, 0.01 μg. (**B**) Partial digestion controlled by reaction duration. Lane M1, Fermentas 100 bp DNA Ladder (selected bands marked); lane M2, Fermentas 1 kb DNA Ladder (selected bands marked); DNA digested with 5 μg (40 pmols) of TaqII at 65°C in the presence of SIN/DMSO (Methods): lane 1, 16 h; lane 2, 8 h; lane 3, 4 h; lane 4, 2 h; lane 5, 1 h; lane 6, 30 min; lane 7, 15 min; lane 8, 7.5 min; lane 9, 3.25 min; lane 10, 1.62 min; lane 11, 0.81 min.

Finally, we practically characterized TaqII affinity star activity and applied it to the construction of representative horse genomic DNA libraries to be used in cloning and construction of intact and truncated butyrylcholinesterase gene variants and domains (manuscript in preparation). This was accomplished by preparation of a BAC library from horse liver genomic DNA, which was created with TaqII/SIN/DMSO-derived inserts in the F factor-based BAC vector pBeloBAC11. Interestingly, bands superimposed over the DNA smear were observed during horse liver genomic DNA digestion (Figure 7C, 8A,B). Because the horse genome is approximately 1000 times larger than the two model bacterial genomes we digested under the same conditions (Figure 7A,B,C), it is technically unlikely that these bands corresponded to enzyme recognition site bias on the eukaryotic DNA; this would be below the detection limit of the ethidium-bromide stained agarose gels. The superimposed bands instead represent repetitive DNA sequences or structural genomic DNA variations detected using TaqII/SIN/DMSO digestion (Figure 8A,B). Although evaluation of this interesting aspect is beyond the scope of this paper, it may prove useful for analysis and detection of such sequences repeats and structural variations.

Variations in reaction duration and enzyme quantity had no effect on the partial digestion pattern detected. For convenience short reaction times with high enzyme concentrations would therefore be preferable for library preparation (Figure 8B). The resulting BAC library contained over 200,000 clones; insert sizes in 110 randomly chosen clones ranged from 7 to 150 kb, with inserts missing from fewer than 5% (manuscript in preparation). Vector-insert junction sequencing of 10 clones (20 junctions) confirmed the TaqII/SIN/DMSO affinity star recognition sites that were systematically evaluated using bacteriophage λ DNA (Figure 6).

**Figure 9:**
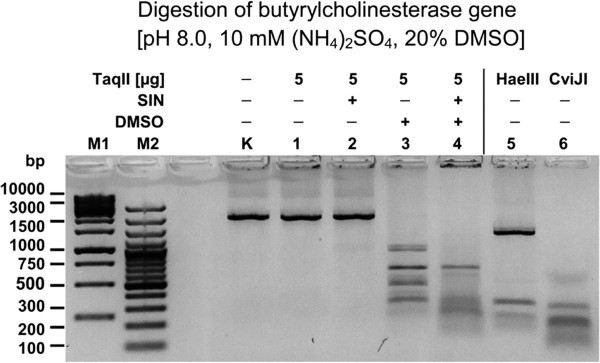
**Comparative digestion of the PCR amplified horse butyrylcholinesterase gene with frequently cleaving REases.** TaqII affinity star cleaved 1 μg horse butyrylcholinesterase gene DNA (1841 bp) was electrophoresed on 1.5% agarose/TBE gel. Cleavage was carried out at 65°C for 16 h with 5 μg (40 pmol) of enzyme in 50 μl of reaction volume. Lane M1, Fermentas 1 kb DNA Ladder (selected bands marked); lane M2, Fermentas 100 bp DNA Ladder (selected bands marked); Lane K, undigested DNA; lanes 1–4, DNA digested with TaqII (Methods): lane 1, without SIN and DMSO; lane 2, with SIN, no DMSO; lane 3, no SIN, 20% DMSO; lane 4, with SIN, 20% DMSO; lane 5, DNA digested with HaeIII (5 units); lane 6, DNA digested with CviJI (0.25 unit).

The cDNA from total horse liver mRNA was subjected to PCR using butyrylcholinesterase-specific primers. The resulting 1841-bp DNA fragment was comparatively digested with three frequently cleaving REases: our novel TaqII/SIN/DMSO tool and two enzymes commonly used for library preparation, HaeIII and CviJI (Figure 9). The digestion fragment pools generated with TaqII/SIN/DMSO were similar in size to those obtained with CviJI (Figure 9; lanes 4 and 6) but smaller than those obtained with HaeIII (5′-GGCC-3′ recognition site) (Figure 9; lane 5). Because substrate DNA was relatively short, discrete bands were also observed especially with HaeIII, which had the longest recognition site. CviJI recognizes 5′-RGCY-3′ sites, which are equivalent to the statistical 3-bp recognition site. Consequently, the calculated 2.9 bp combined recognition site for TaqII/SIN/DMSO is in very good agreement with experimental data, as confirmed by two independent methods: bacteriophage λ shotgun library insert-vector junctions analysis and direct DNA digestion analysed on electrophoretic gels. It is important to note that neither SIN nor DMSO alone trigger the TaqII recognition site transition from 6 bp to 2.9 bp. Apparently, by sufficiently relaxing the tertiary TaqII protein structure, DMSO greatly enhances functional substitution of SAM, bound to the allosteric effector protein pocket, by SIN. Interestingly, one of the TaqII/SIN/DMSO recognition sites listed in Figure 6 is the canonical recognition variant 5′-CACCCA-3′ [28], which is not cleaved by recombinant TaqII in the absence of SIN and DMSO [29].

Small differences observed on the gel between the CviJI and TaqII/SIN/DMSO fragment size distributions may be due to different frequencies of their recognition sites on the butyrylcholinesterase-specific PCR fragment as well as the tendency of TaqII REase to cleave DNA incompletely, as shown previously [28,29]. The tendency toward partial digestion exhibited by TaqII/SIN/DMSO is of practical use in genomic and biotechnology research, however, as it simplifies and allows for greater control of partial DNA digestion during construction of libraries or gene deletion derivatives. The products of such controlled partial digestions of butyrylcholinesterase-specific PCR fragments were cloned and aided the construction of biologically active horse butyrylcholinesterase enzyme (not shown), which was subsequently used in other biotechnological applications (manuscript in preparation).

## Conclusions

In this study TaqII bifunctional REase was shown to change specificity in the presence of a cofactor analogue, SIN (enhanced by DMSO). It was transformed from a 6-bp recognition site enzyme to one of combined 2.9-bp specificity, thus radically increasing cleavage frequency from 4096 bp to 57.7 bp. We found that the addition of DMSO to the SIN-stimulated TaqII reaction had a synergistic enhancing effect, with neither DMSO nor SIN alone effecting complete and maximum specificity transition. TaqII/SIN/DMSO recognition sequence specificity included 70 truncated variants that were 1–2 bp different from the canonical 6-bp recognition site; TaqII/SIN/DMSO DNA cleavage thus does not require the presence of a canonical TaqII recognition site. TaqII affinity star specificity was used to develop a new genomic tool for representative library generation, with its usefulness demonstrated by construction of horse genomic and butyrylcholinesterase gene deletion derivative libraries.

This ultra-frequent DNA cutter also has potential application to other DNA manipulation methods, including ultrasensitive DNA labelling/amplification, high resolution restriction mapping, RFLP, single-copy genes amplifications, metagenomics, and detection/identification of pathogenic microorganisms without culturing.

## Methods

### Bacterial strains, plasmids, media and reagents

We cloned *taqIIRM* gene in *in E. coli,* and produced the TaqII recombinant protein (manuscript in preparation). *E. coli* DH11S {*mcrA* Δ[*mrrhsdRMS*(rK-, mK+)-*mcrBC*] Δ(*lac-proAB*) Δ(*recA1398*) *deoR*, *rpsL*, *srl-thi*, *supE*/*F*′ *proAB*+ *lacI*Q*Z*Δ*M15*} (Life Technologies, Gaithersburg, MD, USA) was used for the transformation of ligation mixtures and DNA propagation. Bacteria were grown in 2× yeast extract/tryptone (YT). For protein expression, bacteria were cultivated in Terrific Broth (TB) medium [17]. *E. coli* K12 ER2420 {F^-^*ara-14 leu fhuA2* Δ*(gpt-proA) 62 lacY1 glnV44 galK2 rpsL20 xyl-5 mtl-1* Δ(*mcrC-mrr*)_HB101_} carrying pBeloBAC11 plasmid was from New England Biolabs (Ipswich, MA, USA).

The DNA purification kits were from A&A Biotechnology (Gdansk, Poland), the T4 DNA ligase from Epicentre Biotechnologies (Madison, USA), the PCR 20-bp Low Ladder from Sigma-Aldrich Poland, the GeneRuler™ 100 bp and 1 kb DNA Ladders from Thermo Fisher Scientific/Fermentas (Vilnius, Lithuania), the Taq DNA Polymerase, λ DNA, SmaI and vector pUC19 from Vivantis (Shah Alam, Malaysia), and the vector pBeloBAC11 from New England Biolabs. The DNA sequencing and PCR primer synthesis were performed at Genomed (Warsaw, Poland). All other reagents were purchased from Sigma-Aldrich (St. Louis, MO, USA).

### PCR fragment DNA cleavage assay

To examine the details of TaqII DNA cleavage pattern in the presence of SIN and DMSO two PCR fragments were used.

The PCR (WT) fragment (390 bp), used as a control substrate DNA, devoid of recognition sequences for TaqII enzyme, was amplified from pBR322 plasmid DNA using Taq DNA polymerase and a pair of primers: FWT 5′-CTCGACCTGAATGGAAGCCG-3′ and RWT 5′-GGTGCAGGGCGCTGACTTCC-3′ (Figure 1A) [32].

For the second PCR (SINGLE) substrate (390 bp), a newly redefined canonical 5′-GACCGA-3′ site [29] was introduced to the PCR (WT) DNA fragment by site-directed mutagenesis, using the extended forward primer 5′-CTCGACCTGAATGGAAGCCGGCGGCACCTCGCT**gACcGA**TTCACCACT-3′ (the nucleotides changed as compared to PCR (WT) are written in small letters; the TaqII site is in bold and underlined). The resulting PCR fragment contained an asymmetrically located single site (→) for TaqII (Figure 1B).

TaqII cleavage of PCR substrates was carried out in reaction buffers (selected for minimizing or enhancing affinity star activity, based initially on the criteria of pH and salt concentration [29]) in the presence or absence of saturating 100 μM SIN concentrations and various concentrations of DMSO. The reaction mixtures containing 1.2 pmol of the TaqII recognition site and 6 pmol recombinant TaqII (1 μg protein) were incubated for 16 h at 65°C. The protein to DNA recognition site molar ratio was approximately 5:1 and the reaction volume was 50 μl. Following digestion, proteinase K to 100 μg/ml, sodium dodecyl sulphate to 0,5%, EDTA to 5 mM were added [17] to the solution, and the incubation was continued for a further 3 h at 55°C. The mixtures were phenol/chloroform-extracted and the digested DNA was ethanol-precipitated. Finally, the DNA precipitate was collected by centrifugation and dissolved in 10 mM. Tris–HCl, pH 8.0, at 25°C. An analogous procedure was employed to digest the 1841-bp PCR fragment, containing the coding region of the horse butyrylcholinesterase gene, obtained from the total cDNA template. The primers used were as follows: 5′-TCAGTATGCAGAGCTGGGGTACAATC -3′ (forward) and 5′- GGTACACACGCGCCGTCTTTG -3′ (reverse). PCR products, genomic DNA, REase digestion products were analysed using agarose or polyacrylamide gel electrophoresis in Tris-Borate-EDTA (TBE) buffer [17], followed by visualization either with ethidium bromide or Sybr Green I and spectrophotometric quantification using the NanoDrop 1000 Spectrophotometer (Thermo Scientific).

### λ DNA cleavage assay, shotgun fragment generation and determination of TaqII affinity star recognition as well as cleavage sites in the presence of SIN and DMSO

Cleavage was carried out in the reaction buffer finally optimized for the intertwined action of reaction conditions, resulting in synergistic maximum TaqII affinity star activity (40 mM Tris–HCl, pH 8.0, at 65°C, 10 mM MgCl_2_, 10 mM (NH_4_)_2_SO_4_, 1 mM DTT, BSA 100 μg/ml, 100 μM SIN, 20% DMSO) at 65°C. The control reaction proceeded in the absence of SIN or DMSO. The reaction volume of 50 μL contained 0.32 pmol recognition sites (1 μg of λ DNA) and 16 pmol recombinant TaqII protein (2 μg of protein). The molar ratio of protein to DNA recognition sites was approximately 50:1. After 16 h, the digestion was quenched with phenol/chloroform, and DNA was ethanol-precipitated from the aqueous phase. The DNA precipitate was collected by centrifugation and dissolved in 10 mM Tris–HCl, pH 8.0, at 25°C. The DNA samples were treated with T4 DNA polymerase in the presence of dNTP. The concentration of TaqII given here refers to the monomeric form of protein Mr 120,000. The TaqII affinity star recognition site and cleavage positions were established by shotgun cloning and sequencing of the digestion products of λ DNA. The TaqII/SIN/DMSO-generated restriction fragment ends were blunted with T4 DNA polymerase in the presence of dNTPs, cloned into the SmaI site of the pUC19 vector, transformed into *E. coli* DH11S, and plated onto X-gal/IPTG plates [17]. Plasmid DNA was isolated from white colonies, and the multiple fragment/vector junctions were sequenced. The sequence data obtained were analyzed using ABI Chromas 1.45 software (Perkin Elmer Applied Biosystems, Monza, Italy) and DNASIS 2.5 software (Hitachi Software, San Bruno, CA, USA). The same procedure was used to clone partial digestion fragments of PCR from cDNA, coding for horse butyrylcholinesterase, except that a dedicated expression vector was used for ligation (manuscript in preparation).

### Genomic DNA purification and cleavage assays

Bacterial genomic DNA was purified from *T. thermophilus* and *E. coli* DH5α using the Genomic Mini DNA purification kit (A&A Biotechnology).

Eukaryotic genomic DNA was isolated from horse liver, obtained from a local horse butchery, as described [39]. Care was taken to avoid hydrodynamic sharing and to obtain a DNA molecule population dominated by >50 kb genomic fragments. An additional step was added as a final clean-up of the isolated DNA, employing digestion with RNase A (100 μg/ml) for 2 h at 37°C followed by phenol–chloroform extraction and ethanol precipitation [17]. TaqII/SIN/DMSO cleavage of horse liver, *T. thermophilus* and *E. coli* DH5α genomic DNAs was carried out as described for λ DNA with the following modifications.

*Bacterial genomic DNA*: the amount of TaqII protein added to the reaction mixture was 2 μg and the amount of substrate DNA was 500 ng. The molar ratio of protein to DNA recognition sites could not be precisely calculated. However, in view of the identical % GC content in *E. coli* DNA and in λ DNA; given the large genome size, averaging TaqII recognition sites distribution, the expected cleavage products are expected to be of similar length. Analogously, the cleavage of *T. thermophilus DNA*, performed under conditions favouring canonical TaqII recognition site only (5′-GACCGA-3′: 75% GC), should result in smaller final fragments distribution due to the high 69.4% GC content. However, 70 variants of TaqII/SIN/DMSO combined recognition sites are averaging GC content bias, thus the app. 50:1 molar ratio was assumed to be similar as well. *Horse liver genomic DNA*: 2 μg of TaqII protein were added to the reaction mixture; 500 ng of substrate DNA was used. *Partial cleavage of horse liver genomic DNA:* Partial digestion of 1 μg genomic DNA was carried out for 3 h with decreasing amounts of TaqII protein (from 5 μg to 10 ng) or with a fixed amount of TaqII (5 μg; 40 pmol) but for decreasing digestion times (from 16 h to less than 1 min) in the presence of 100 μM SIN and 20% DMSO for the construction of the BAC DNA library, which was subsequently used to select the butyrylcholinesterase gene (manuscript in preparation). The preferred conditions for partial digestion were determined either as the amount of TaqII protein needed or as the digestion timing (Figure 8A,B). The enzyme concentration/timing that generated fragments with a majority of over 10 kb was selected for partial scale digestions and used for subsequent cloning procedures.

### pBeloBAC11 vector preparation and library construction

Vector pBeloBAC11 (7.507 kb) in *E. coli* strain K12 was streaked out onto an LB plate containing 40 μg/ml chloramphenicol (CM), X-GAL and IPTG and grown at 37°C overnight. A single blue colony was used to inoculate 4 1 of LB media, containing 40 μg/ml CM, grown overnight at 30°C, and pBeloBAC11 DNA was extracted from the resulting cells by alkaline lysis, phenol-chloroform treatment and ethanol precipitation [17]. The pBeloBAC11 [40] was digested with SphI, DNA ends were repaired with T4 DNA polymerase in the presence of dNTPs and dephosphorylated with calf intestinal alkaline phosphatase [17]. The digested vector DNA was purified, precipitated and dissolved in 10 mM Tris–HCl, pH 8.0. The horse genomic DNA was partially digested with TaqII/SIN/DMSO, followed by DNA end blunting with T4 DNA polymerase in the presence of dNTPs and ligation into the previously prepared pBeloBAC11 vector at an approximate molar ratio of 1:5, with T4 DNA ligase at 16°C for 12 h. DNA from the ligation mixture was purified and used for electroporation of *E. coli* DH11S. The bacteria were grown on X-gal/IPTG plates [17]. Colourless clones were inoculated in 5 ml of LB broth containing 40 μg/ml of CM and grown at 37°C overnight. The cells were harvested and DNA was isolated with the alkaline lysis method [17]. The fragment/vector junctions were sequenced.

## Competing interests

The authors declare that they have no competing interest.

## Authors’ contributions

AZS conceived the project, coordinated its execution, participated in the design and interpretation of all the experimental analyses, performed some experiments, prepared the figures and drafted the manuscript. OZ performed most of the experiments. JJ prepared the horse liver genomic DNA, butyrylcholinesterase cDNA and PCR, and participated in the design and interpretation of some experimental analyses. PMS participated in the design and interpretation of the experiments, coordinated the execution of the project and drafted the manuscript. All the authors read and approved the final manuscript.
